# A cross-cultural exploration of compassion, and facilitators and inhibitors of compassion in UK and Sri Lankan people

**DOI:** 10.1017/gmh.2022.10

**Published:** 2022-02-23

**Authors:** Lasara Kariyawasam, Margarita Ononaiye, Chris Irons, Sarah E. Kirby

**Affiliations:** 1 Department of Psychology, University of Southampton, Southampton, UK; 2 Balanced Mind, London, UK

**Keywords:** Compassion, cross-cultural, facilitators, inhibitors, society, Sri Lankan, UK

## Abstract

**Background:**

Practising compassion has shown to increase well-being and reduce distress in people across cultures. However, very little research has explored cultural differences in different facets of compassion with a dearth of research evident especially in the Asian context. Several inhibitors and facilitators of compassion have been identified although the nuances of cultural differences of these remain unexploited. This study aimed to discover cross-cultural similarities and differences of the levels of compassion, facilitators and inhibitors of compassion between Sri Lankan and UK people.

**Methods:**

A cross-sectional, questionnaire-based quantitative research was conducted among 149 Sri Lankan and 300 UK participants. Individual predictors (such as fears of compassion, self-reassurance, external shame, social safeness and pleasure, depression and anxiety) were also explored in relation to compassion, compassion to others, and compassion from others in each group.

**Results:**

The results indicated that Sri Lankan participants were more self-reassured and self-compassionate and self-identifying as a Buddhist predicted higher self-compassion, when compared to UK participants. However, Sri Lankan participants reported higher levels of external shame and fear of compassion not just towards themselves, but also towards and from others, indicating difficulty in engaging compassionately with others. In contrast, UK participants reported higher social safeness, indicating that they were more likely to feel safe and soothed by the society than the Sri Lankan participants.

**Conclusions:**

Society plays a pivotal role in shaping one's experiences of compassion. This study suggests that specific cultural and social factors should be considered when implementing Western compassionate approaches to non-Western settings.

Compassion is ‘a sensitivity to the suffering of self and others, with a deep wish and commitment to relieve the suffering’ (Gilbert, n.d., p. 22). The underlying motive is to collate our emotions into a helpful balance that enhances our sense of well-being (Gilbert, 2014*a*). Practising compassion towards others and oneself has shown promising potential for increasing well-being (Lutz *et al.*, 2004). Developing compassionate thoughts towards the self, others and even strangers, has increased positive emotions, social support and mindfulness (Fredrickson *et al*., 2008), and decreased depression and anxiety (MacBeth and Gumley, 2012). Despite the perceived benefits and increased interest in compassion practice, there is a dearth of research investigating the cross-cultural differences of compassion and the multifaceted factors affecting it (Montero-Marin *et al*., 2018). Therefore, this study attempted to explore compassion, and facilitators and inhibitors of compassion among people in Sri Lanka, a collectivistic Asian culture, in comparison with a group of UK people.

## Theoretical perspective of compassion

Gilbert (2014*b*, 2016) developed social mentality theory (SMT), with the notion that compassion is an evolved care-based motivational system, known as a social mentality, which originally evolved to regulate distress in parent–infant relationships. The SMT is underpinned by evolutionary psychology, neurophysiology (Porges, 2007), attachment theory (Bowlby, 1982) and Buddhism (Wong, 2021). It emphasises that compassion activates the motivation to pay attention to suffering to make sense of it, and the ability to relieve and prevent that suffering (Gilbert, 2000). The social mentality of compassion comprises six essential competencies that are related to sensitivity, sympathy, distress tolerance, empathy, non-judgement and care for well-being (Gilbert, 2009). These competencies flow across three directional paths, known as the three flows of compassion, which are *compassion to others*, *compassion from others* and *self-compassion* (Gilbert, 2014*a*). Based on the aforementioned theory, Gilbert (2009) introduced compassion focused therapy (CFT), to treat people experiencing psychological issues that involve high levels of shame and criticism, by cultivating compassion across the three flows.

Gilbert (2005*b*) used a tripartite model to conceptualise psychopathology. According to this model of affect regulation, three systems interact to regulate signals of threat, resources/incentives and affiliation/soothing, which trigger the negative affect, high arousal positive affect and social safeness, respectively. This model explains how various psychosocial vulnerabilities can be understood using the interplay between these three regulatory systems (Gilbert, 2005*a*, 2015). Whilst an overactive threat system is found to inhibit compassion cultivation, the soothing system holds the capacity to suppress the threat system and facilitate the manifestation of compassion (Gilbert *et al*., 2008).

## Inhibitors and facilitators of compassion

According to Gilbert (2007, n.d., 2014*b*), attachment insecurities, neglect, abuse or emotional conflicts with significant others generate fear reactions, such as avoidances and resistances that inhibit compassion. Such experiences pose a vulnerability to self-criticism, which hinders compassion cultivation (Rector *et al*., 2000), and acts as a pervasive element of shame and psychopathology (Gilbert and Irons, 2005). Studies have found that the conceptualisation of shame may differ between cultures (Mesquita, 2001), with shame being an internal, self-directed construct in individualistic cultures and an external construct, which relates to how a person exists in the minds of others and their judgements (Gilbert, 1998), in collectivistic cultures such as the Asian countries. Self-criticism, fears of compassion and experiencing shame are found to positively correlate with depression (Gilbert *et al*., 2011, 2014) and anxiety (Gilbert *et al*., 2014; Hermanto *et al*., 2016). Thus, psychopathology, including depression and anxiety is believed to stem from an over-activation of the threat system and an under-activation of the soothing system making it difficult for one to experience compassion (Gilbert *et al*., 2014).

On the contrary, the soothing system seeks signals of care, warmth and affiliation, and arouses calmness and reassurance (Gilbert *et al*., 2008). Therefore, in the presence of *social safeness*, the warm, calming experience of feeling cared about, reassured by, and connected to others in the society, people are more likely to generate warm affiliative feelings such as compassion (Gilbert *et al*., 2009). Self-reassurance is another factor that activates the soothing system and facilitates compassion. In fact, the ability to self-reassure and recognise one's strengths during suffering has reduced depression in clinical and non-clinical groups (Castilho *et al*., 2013). Studies have found that whilst self-criticism inhibits compassion and correlates with depressive symptomatology, higher ability to self-reassure could weaken this relationship between self-criticism and depression (Petrocchi *et al*., 2019). This indicates that although it has been discovered that external shame and attachment insecurities can suppress one's compassion (Gilbert and Irons, 2005), a soothing-affiliation system with others can increase compassion across all three flows (Gilbert, 2005*a*).

## Compassion across cultures

Despite the increased interest in compassion research and the evidence supporting the benefits of compassion practice, most studies are limited to Western countries (Neff *et al*., 2008; Sinclair *et al*., 2016). Application of Western models to non-Western societies is challenging, as compassion is a context-dependent construct influenced by group norms, cultural practices and values (Gilbert *et al*., 2011). Whilst compassion is seen as universal, cross-cultural differences have been identified in various facets of compassion (Birkett, 2013), such as the six compassion competencies (Gilbert, 2009), and inhibitors and facilitators of compassion (Steindl *et al*., 2020). Eastern collectivistic societies such as Sri Lanka (Pathirana, 2016) are appreciative of devotion and concerns for others (Triandis, 1993), and may show more compassion to others than the Western societies (Steindl *et al*., 2020). Thus, it seems fair to propose that people's underlying motivations and views of compassion may vary cross-culturally (Cheon *et al*., 2011).

Neff (2011) viewed self-compassion as an Asian construct due to compassion being broadly discussed in Buddhism, a religion that is primarily followed by Asian people (Prebish and Baumann, 2002). From a Buddhist standpoint, compassion is the desire to free all people from suffering (Davidson and Harrington, 2002), and Buddhist practices such as loving-kindness and mindful meditation (Leighton, 2012) promote compassion cultivation (Lama and Vreeland, 2008). Thus, one would expect Buddhist followers to be affluent in compassion. In support, a study conducted in the USA where the majority of the participants self-identified as Caucasian found that participants practising Buddhist meditation were more self-compassionate than college undergraduates and older adults recruited from the wider community (Neff and Pommier, 2013).

In contrast, a study that explored practising Buddhists in a Japanese collectivistic country, where people's lifestyle is influenced by high levels of social interconnectedness (Neff *et al*., 2008), discovered that Japanese participants had low self-compassion and high self-criticism when compared to the USA participants (Kitayama and Markus, 2000). The social pressure to abide by cultural norms in Japan may explain their low self-compassion (Neff *et al*., 2008). Thus, despite the strong Buddhist influence of compassion, cultural differences may tentatively explain why Asian Buddhist people living in Western countries indicated higher self-compassion (Neff and Pommier, 2013). In support, Wong (2021) emphasised that the lives of several Asian people living in Asian countries are controlled by external forces, pain and tragedy that are beyond their control, which may explain their general lack of self-compassion. Furthermore, Asian Confucian cultures, such as Taiwan, where self-improvement is determined by shame, judgement and threatened isolation indicated higher self-criticism rather than self-compassion. In the same study, however, Thai participants (a Buddhist-influenced culture) were more self-compassionate than the American and Taiwanese participants. The collectivistic social dynamic in cultures such as Sri Lanka is found to inhibit people from receiving compassion from themselves and others (Montero-Marin *et al*., 2018; Steindl *et al*., 2020; Kariyawasam *et al*., 2021), due to eastern cultural norms discouraging help-seeking behaviour, as seeking help is considered as a failure that brings shame to one and those around oneself (Kee, 2004). Thus, it seems fair to propose that whilst the Buddhist religion encourages compassion, the collectivistic cultural dynamic may be a factor that inhibits people's compassionate experiences. However, only a few studies have looked at self-compassion in a cross-cultural Asian context (Neff *et al*., 2008; Birkett, 2013), implying the need for further research.

So far, studies exploring the three flows of compassion in the Asian context remain to be very limited (Asano *et al*., 2020). It is also noteworthy that several Asian people feel that Western theories are only applicable to people living in the West, as they believe that compared to Westerners, they have been through, and continue to face, more tragedy and pain in their daily living (Wong, 2021). For example, Sri Lanka is a multi-ethnic, multi-cultural collectivistic South Asian island, where almost 70% of the population practice Buddhism (De Zoysa, 2021). Sri Lankans have, however, experienced several catastrophes such as a civil war and tsunami over the past few decades, and report high rates of grief, domestic violence, learned helplessness, alcohol abuse, self-harming and attempted suicides (World Health Organisation, 2018), depression, anxiety and post-traumatic stress disorder (Gunaratnam *et al*., 2003). In view of this, it is presumed that Sri Lankan people may benefit from compassion cultivation.

## Rationale for the current study

In consideration of the aforementioned, including the proposal that compassion is at least partially determined by culture, cross-cultural explorations remain at an infancy stage (Montero-Marin *et al*., 2018). Furthermore, as one's level of compassion is determined by specific cultural practices that are more nuanced than a simple East-West contrast (Neff *et al*., 2008), there is an apparent research gap on cross-cultural compassion.

This study aimed to compare the three flows of compassion (self-compassion, compassion to and from others) between Sri Lankan and UK participants, to explore cross-cultural similarities and differences in the compassion constructs. Additionally, this research investigated which of the inhibitors of compassion (of fears of compassion, self-criticism and external shame), and facilitators of compassion (of self-reassurance and social safeness) and psychopathology (depression and anxiety) have the biggest impact on predicting each of the three flow of compassion within a cross-cultural perspective. Due to the scarcity of cross-cultural studies and ambiguity of the theoretical associations of the concepts discussed above (Gilbert, 2005*a*; Neff *et al*., 2008; López *et al*., 2018), no firm directional hypotheses were constructed.

## Method

### Research design

This study used a cross-sectional, between-participants, questionnaire-based exploratory quantitative research design.

### Participants

Participants were either UK or Sri Lankan nationals, and at least 18 years old. Participants were required to self-identify their nationality, and all participants had to be fluent in English language. The final sample comprised 300 UK and 149 Sri Lankans.

### Measures

Demographic information on age, gender, religion and nationality was obtained. In addition, the following measures were administered in English.

Compassionate Engagement and Action Scales (CEAS: Gilbert *et al*., 2017) measured participants' compassionate engagement and action in the three flows: self-compassion (engagement *α* = 0.77, action *α* = 0.90), compassion to others (engagement *α* = 0.90, action *α* = 0.94) and compassion from others (engagement *α* = 0.89, action *α* = 0.91), with 13 items measuring each flow. Answers ranged on a Likert-scale from 1 (never) to 10 (always).

Fears of Compassion Scales (FOCS: Gilbert *et al*., 2011) measured the fears of self-compassion (15 items), compassion from others (13 items) and compassion to others (10 items) on a 5-point Likert scale from 0 (don't agree at all) to 4 (completely agree). This scale indicated a good reliability for all three items (*α* *=* 0.85 for fear of self-compassion, *α* *=* 0.87 for fear of compassion from others and *α* *=* 0.78 for fear of compassion to others).

The Forms of Self-Criticising/Attacking and Self-Reassuring Scale (FSCRS: Gilbert *et al*., 2004) assessed self-criticism and self-reassurance on three dimensions: inadequate self, hated self and reassured self. It is a 22-item Likert scale ranging from 0 (not at all like me) to 4 (extremely like me) and is designed to measure people's thoughts and feelings about themselves in times of distress. A good reliability has been reported for all three dimensions (e.g. *α* *=* 0.90 for inadequate self, *α* *=* 0.86 for hated self, and *α* *=* 0.86 for reassured self).

The Others as Shamer Scale (OAS: Allan *et al*., 1994) tested participants' perception of how others see them, referred to as external shame. This is an 18 item, 5-point Likert scale from 0 (never) to 4 (almost always), with a high internal consistency of *α* *=* 0.96.

The Social Safeness and Pleasure Scale (SSPS: Gilbert *et al*., 2009) measured the extent to which people perceive their social world as safe and warm. This 12-item scale ranging from 0 (almost never) to 4 (almost all the time) has acquired a high alpha value of *α* *=* 0.92.

Finally, anxiety and depression were assessed using the Generalised Anxiety Disorder-7 scale: (GAD-7: Spitzer *et al*., 2006), and Patient Health Questionnaire (PHQ-9: Kroenke *et al*., 2001), respectively. Both scales are scored on a Likert scale from 0 (not at all) to 3 (nearly every day) and have obtained an excellent internal reliability of *α* *=* 0.89 (Löwe *et al*., 2008).

### Procedure

This study was approved by the Ethics Committee of the University of Southampton (ID: 52533.A1). Participants were conveniently recruited from multiple online platforms (e.g. Facebook and Linkedin). A series of questionnaires including a demographic questionnaire, CEAS, FOCS, FSCRS, OAS, SSPS, GAD-7 and PHQ-9 were presented respectively, after obtaining participants' informed consent.

### Data analysis plan

Analyses of covariance (ANCOVA) tested the first aim to determine whether there were differences between the Sri Lankan and UK groups in their three flows of compassion and inhibitors and facilitators (using scores of FOCS, FSCRS, OAS, SSPS, PHQ-9 and GAD-7), controlling for age and gender. Six hierarchical multiple linear regressions (one for each flow of compassion in each country) were then conducted between the two groups, to test the second aim and exploring similarities and differences in the predictors of compassion (scores of FOCS, FSCRS, OAS, SSPS, PHQ-9 and GAD-7 as predictors). In the first block, religion, age and gender were entered so that the demographics could be controlled for. Depression and anxiety scores were controlled in the second block. The final block contained all the controlled variables and the remaining scales (FOCS, FSCRS, OAS and SSPS).

## Results

There were more females (*n* = 97, 65% Sri Lankan; *n* = 272, 91% UK) compared to males (*n* = 52, 35% Sri Lankan; *n* = 27, 9% UK) in both samples [χ^2^= (1, *N* = 448) = 45.819, *p* < 0.001]. Sri Lankans were significantly older than the UK participants [*t*(447) = 6.784, *p* < 0.05], with the ages ranging from 18 to 50 years in Sri Lankans (*M* = 24.82, s.d. = 4.70) and 18–62 years in the UK participants (*M* = 20.95, s.d. = 6.11). Chi-squared for religion was significant, χ^2^ = (7, *N* = 449) = 333.320, *p* < 0.001. Majority of the Sri Lankans were Buddhists (74%) and the majority of the UK sample identified themselves as atheists (62%). As the significant differences in the demographic factors (religion, age and gender) could potentially affect the overall results, these factors were controlled.

### Aim 1: testing compassion and, inhibitors and facilitators of compassion between Sri Lankan and UK samples

ANCOVA tests were conducted to determine if there would be a difference between the Sri Lankan and UK groups on their levels of compassion and associated inhibitors and facilitators, controlling for the demographics (see Table 1). The Sri Lankan group reported higher self-compassion and self-reassurance compared to the UK group, although of inhibitors, they also reported higher fears across all three flows of compassion and perceived external shame. In contrast, the UK group indicated greater levels of social safeness. No significant differences were found for compassion to and from others, and depression and anxiety between the two groups.

**Table 1.** Means, standard deviations and ANCOVA results

Measure (scale range)	SL (*N* = 149)	UK (*N* = 300)	Age covariate			Gender covariate			Nationality		
	*M* (s.d.)	*M* (s.d.)	*F*	*p*	sr^2^	*F*	*p*	sr^2^	*F*	*p*	sr^2^
**Self-compassion** (**10–100)**	66.64 (12.87)	61.22 (13.15)	0.65	0.422	0.001	2.07	0.151	0.005	12.91	**0**.**000**	0.028
Compassion from others (10–100)	60.76 (16.72)	64.11 (15.09)	21.44	**0.000**	0.046	3.99	**0.046**	0.009	0.01	0.939	0.000
Compassion to others (10–100)	75.97 (12.66)	79.44 (10.02)	0.28	0.599	0.001	12.12	**0.001**	0.027	3.10	0.079	0.007
**Fear of compassion to others** (**0–40)**	23.03 (6.99)	18.10 (7.22)	0.63	0.427	0.001	0.00	0.971	0.000	37.01	**0.000**	0.077
**Fear of compassion from others** (**0–52)**	24.12 (10.23)	16.21 (10.00)	5.69	**0.017**	0.013	0.29	0.590	0.001	44.20	**0.000**	0.091
**Fear of self-compassion** (**0–60)**	22.10 (17.52)	17.52 (13.52)	4.48	**0.035**	0.010	0.58	0.446	0.001	4.89	**0.028**	0.011
**Reassured self** (**0–32)**	20.44 (18.28)	18.28 (6.44)	0.85	0.357	0.002	4.29	**0.039**	0.010	7.10	**0.008**	0.016
Inadequate self (0–36)	21.88 (8.11)	22.24 (7.68)	0.60	0.437	0.001	3.19	0.075	0.007	0.01	0.924	0.000
Hated self (0–20)	6.31 (5.27)	5.74 (5.23)	2.06	0.152	0.005	0.01	0.908	0.000	0.35	0.554	0.001
**Others as shamer** (**0–72)**	35.50 (18.53)	26.68 (14.91)	1.08	0.299	0.002	3.51	0.062	0.008	27.55	**0.000**	0.058
**Social safeness** (**11–55)**	36.30 (9.72)	39.61 (9.10)	7.79	**0.005**	0.017	0.15	0.699	0.000	5.29	**0.022**	0.012
Anxiety (0–21)	10.69 (5.15)	9.60 (5.30)	8.02	**0.005**	0.018	4.81	**0.029**	0.011	3.11	0.078	0.007
Depression (0–27)	11.36 (6.95)	9.77 (6.38)	9.49	**0.002**	0.021	0.53	0.465	0.001	2.43	0.120	0.005

Variable that has a significant *p* value of (<.05) has been presented in bold.

The results were in line with a cross-cultural study that found that Singaporean participants were more self-compassionate compared to the Australian participants (Steindl *et al*., 2020). They also found that although people from the collectivistic Singaporean background were expected to show higher compassion to others, the Australian participants were not only more likely to receive compassion from others, but also more compassionate to others than the Singaporeans. Similarly to the current study, Singaporean participants also indicated a greater fear of compassion towards others (Steindl *et al*., 2020). These distinctions will be discussed in the discussion section.

### Aim 2: predictors of the three flows of compassion in the UK and Sri Lankan samples

#### Predictors of self-compassion in Sri Lankan and UK participants

A hierarchical multiple linear regression was carried out to predict self-compassion based on the subscales of FOC, FSCRS, OAS and SSPS scales whilst controlling for religion, age, gender, anxiety and depression. In the Sri Lankan participants, a significant regression equation [*F*(14, 134) = 8.88, *p* < 0.001] was resulted in an *R*^2^ of 0.48 (Table 2). Following Buddhism, being older in age, high self-reassurance and lack of fear of self-compassion predicted greater self-compassion. Results implied that Sri Lankan participants who were less fearful of showing self-compassion were more self-reassured and therefore, more self-compassionate.

**Table 2.** Regression results for predictors of self-compassion in Sri Lankan participants

Block 3	*B*	SEB	*β*	*t*	Sig.	*r* zero order	sr^2^	95% confidence interval (CI)
**Buddhist religion**	**6.20**	**2.07**	**0.21**	**3.00**	**0.003**	**0.32**	**0.035**	**2.11 to 10.31**
Atheist religion	−3.22	2.26	−0.10	−1.43	0.155	−0.20	0.008	−7.69 to 1.23
Gender	−1.55	1.86	−0.06	−0.84	0.405	−0.11	0.003	−5.23 to 2.12
**Age**	**0.41**	**0.19**	**0.15**	**2.24**	**0.027**	**0.15**	**0.020**	**0.05–0.79**
Anxiety	−0.32	0.23	−0.13	−1.40	0.163	−0.30	0.008	−0.78 to 0.13
Depression	−0.16	0.22	−0.09	−0.75	0.458	−0.38	0.002	−0.59 to 0.27
FCTO	0.02	0.15	0.01	0.13	0.895	0.02	0.000	−0.27 to 0.31
FCFO	0.18	0.16	0.15	1.18	0.241	−0.27	0.005	−0.13 to 0.50
**FSC**	−**0.24**	**0.12**	−**0.26**	−**2.10**	**0.038**	−**0.37**	**0.017**	−**0.48 to −0.01**
Inadequate self	0.19	0.18	0.12	1.05	0.295	−0.39	0.004	−0.17 to 0.55
**Reassured self**	**0.88**	**0.21**	**0.48**	**4.23**	**0.000**	**0.63**	**0.069**	**0.47 to 1.30**
Hated self	0.19	0.36	0.08	0.55	0.586	−0.47	0.001	−0.51 to 0.90
Others as shamer	0.05	0.09	0.08	0.61	0.540	−0.42	0.001	−0.12 to 0.23
Social safeness	0.15	0.13	0.12	1.21	0.227	0.47	0.006	−0.10 to 0.41

*Note.* sr^2^: small effect size = 0.02, medium effect size = 0.15, large effect size = 0.35. SEB: standard error of B, FCTO: fear of compassion to others, FCFO: fear of compassion from others, FSC: fear of self-compassion.

Variable that has a significant *p* value of (<.05) has been presented in bold.

In the UK participants, a significant regression equation [*F*(14, 284) = 14.73, *p* < 0.001] resulted in an *R*^2^ of 0.42, for self-compassion (Table 3). Higher self-reassurance and external shame predicted self-compassion in UK participants with small–medium and small-effect sizes, respectively.

**Table 3.** Regression results for predictors of self-compassion in UK participants

Block 3	*B*	SEB	*β*	*t*	Sig.	*r* zero order	sr^2^	95% CI
Buddhist religion	0.65	5.38	0.01	0.12	0.904	0.05	0.000	−9.93 to 11.23
Atheist religion	−0.67	0.63	−0.05	−1.06	0.290	−0.08	0.002	−1.91 to 0.57
Gender	−0.80	2.14	−0.02	−0.37	0.709	−0.03	0.000	−5.00 to 3.41
Age	−0.05	0.10	−0.02	−0.47	0.640	−0.10	0.000	−0.25 to 0.16
Anxiety	0.02	0.19	0.01	0.12	0.909	−0.38	0.000	−0.36 to 0.40
Depression	−0.12	0.18	−0.06	−0.65	0.515	−0.44	0.001	−0.47 to 0.24
FCTO	0.09	0.11	0.05	0.82	0.414	−0.06	0.001	−0.12 to 0.30
FCFO	−0.06	0.12	−0.05	−0.52	0.605	−0.38	0.001	−0.29 to 0.17
FSC	−0.12	0.08	−0.12	−1.40	0.162	−0.45	0.004	−0.28 to 0.05
Inadequate self	−0.20	0.15	−0.12	−1.33	0.186	−0.52	0.004	−0.49 to 0.10
**Reassured self**	**0.98**	**0.16**	**0.48**	**6.24**	**0.000**	**0.63**	**0.080**	**0.668 to 1.29**
Hated self	−0.01	0.21	−0.00	−0.03	0.977	−0.48	0.000	−0.43 to 0.42
**Others as shamer**	**0.15**	**0.07**	**0.17**	**2.10**	**0.036**	−**0.39**	**0.009**	**0.01 to 0.28**
Social safeness	0.10	0.10	0.07	0.90	0.367	0.47	0.002	−0.11 to 0.31

*Note.* sr^2^: small effect size = 0.02, medium effect size = 0.15, large effect size = 0.35.

Variable that has a significant *p* value of (<.05) has been presented in bold.

The significant but positive multivariate relationship between self-compassion and higher external shame in UK participants is striking as a positive relationship is inconsistent with the existing literature (Allan *et al*., 1994; Ferreira *et al*., 2013), which strongly reports external shame as an inhibitor of self-compassion. This is also a change in direction from the zero-order correlation between self-compassion and external shame, which was significantly negative, with a medium-large effect size (*r* = −0.39), consistent with the previous literature. Thus, any possible explanations for this significant directional change were further explored. A simple mediation analyses using PROCESS indicated that self-reassurance significantly mediated the relationship between shame and self-compassion. In step 1 of the mediation model, the regression of perceived external shame on self-compassion, ignoring the mediator, was significantly negative, *b* = −0.35, *t*(298) = −7.38, *p* < 0.001. This meant that self-compassion was lower if the perceived shame was high. Step 2 showed that the regression of external shame on the mediator, self-reassurance, was also significantly negative, suggesting that when participants' perceived shame was high, their levels of self-reassurance was low, *b* = −0.27, *t*(298) = −13.81, *p* < 0.001. Step 3 of the mediation process, however, showed that the mediator (self-reassurance), controlling for external shame, was significantly positive, indicating that participants were more self-compassionate, when they were more self-reassured, *b* = 1.23, *t*(297) = 10.80, *p* = 0.000. As a result, step 4 of the analyses revealed that, controlling for the mediator (self-reassurance), external shame was not a significant predictor of self-compassion, *b* = −0.00, *t*(297) = −0.0354, *p* = 0.9718 in the UK participants. Thus, results explained that although higher external shame inhibits self-compassion, the significantly higher levels of self-reassurance in the UK group meant that their self-compassion was high even in the presence of higher external shame.

#### Predictors of compassion to others in Sri Lankan and UK participants

A hierarchical multiple linear regression indicated a significant regression *F*(14, 134) = 3.76, *p* < 0.001, with an *R*^2^ of 0.28, for predictors of offering compassion to others in Sri Lankans (Table 4). Participants with greater fears of self-compassion were less likely to show others compassion, with higher self-inadequacy predicting higher compassion towards others.

**Table 4.** Regression results for predictors of compassion to others in Sri Lankan participants

Block 3	*B*	SEB	*β*	*t*	Sig.	*r* zero order	sr^2^	95% CI
Buddhist religion	4.44	2.40	0.15	1.85	0.067	0.18	0.018	−0.31 to 9.19
Atheist religion	−4.46	2.61	−0.14	−1.71	0.090	−0.17	0.016	−9.63 to 0.71
Gender	−0.47	2.15	−0.02	−0.22	0.826	0.14	0.000	−4.73 to 3.79
Age	−0.43	0.22	−0.16	−1.97	0.051	−0.22	0.021	−0.85 to 0.00
Anxiety	0.34	0.27	0.14	1.27	0.205	0.22	0.009	−0.19 to 0.87
Depression	0.12	0.25	0.07	0.49	0.624	0.09	0.001	−0.37 to 0.62
FCTO	−0.10	0.17	−0.06	−0.61	0.544	−0.04	0.002	−0.44 to 0.24
FCFO	−0.06	0.18	−0.05	−0.35	0.728	−0.10	0.001	−0.42 to 0.30
**FSC**	−**0.32**	**0.14**	−**0.34**	−**2.32**	**0.022**	−**0.14**	**0.029**	−**0.59 to −0.05**
**Inadequate self**	**0.67**	**0.21**	**0.43**	**3.20**	**0.002**	**0.19**	**0.055**	**0.26 to 1.09**
Reassured self	−0.00	0.24	−0.00	−0.01	0.995	0.05	0.000	−0.48 to 0.48
Hated self	−0.40	0.41	−0.16	−0.96	0.338	−0.02	0.005	−1.21 to 0.42
Others as shamer	0.08	0.10	0.12	0.78	0.439	0.08	0.003	−0.12 to 0.28
Social safeness	0.21	0.15	0.16	1.38	0.170	0.09	0.010	−0.09 to 0.50

*Note.* sr^2^: small effect size = 0.02, medium effect size = 0.15, large effect size = 0.35.

Variable that has a significant *p* value of (<.05) has been presented in bold.

In the UK participants, a significant regression [*F*(14, 284) = 2.80, *p* < 0.001] with an *R*^2^ of 0.12 was found for offering compassion to others (Table 5). UK participants were more likely to be compassionate towards others, if they were female, less fearful of offering giving compassion to others, and more anxious.

**Table 5.** Regression results for predictors of compassion to others in UK participants

Block 3	*B*	SEB	*β*	*t*	Sig.	*r* zero order	sr^2^	95% CI
Buddhist religion	−3.55	5.01	−0.04	−0.71	0.479	−0.02	0.002	−13.42 to 6.32
Atheist religion	−0.83	0.59	−0.08	−1.40	0.162	−0.07	0.006	−1.99 to 0.33)
**Gender**	**6.59**	**1.99**	**0.19**	**3.31**	**0.001**	**0.20**	**0.034**	**2.66 to 10.51**
Age	0.12	0.10	0.08	1.26	0.210	0.04	0.005	−0.07 to 0.31
**Anxiety**	**0.37**	**0.18**	**0.20**	**2.05**	**0.042**	**0.10**	**0.013**	**0.01 to 0.72**
Depression	−0.12	0.17	−0.08	−0.71	0.478	0.01	0.002	−0.45 to 0.21
**FCTO**	−**0.28**	**0.10**	−**0.20**	−**2.78**	**0.006**	−**0.23**	**0.024**	−**0.48 to −0.08**
FCFO	−0.10	0.12	−0.10	−0.97	0.335	−0.11	0.003	−0.32 to 0.11
FSC	0.03	0.08	0.03	0.33	0.743	−0.04	0.000	−0.13 to 0.18
Inadequate self	0.04	0.14	0.03	0.29	0.775	0.03	0.000	−0.23 to 0.31
Reassured self	0.10	0.15	0.06	0.66	0.511	0.00	0.001	−0.19 to 0.38
Hated self	−0.04	0.20	−0.02	−0.20	0.840	0.02	0.000	−0.43 to 0.35
Others as shamer	0.034	0.07	0.05	0.53	0.599	0.01	0.001	−0.09 to 0.16
Social safeness	0.00	0.10	0.00	0.00	0.999	0.04	0.000	−0.20 to 0.20

*Note.* sr^2^: small effect size = 0.02, medium effect size = 0.15, large effect size = 0.35.

Variable that has a significant *p* value of (<.05) has been presented in bold.

#### Predictors of compassion from others in Sri Lankan and UK participants

A similar linear regression indicated (Table 6) a significant regression [*F*(14, 134) = 2.73, *p* < 0.001] with an *R*^2^ of 0.22 in Sri Lankan participants. Females were more likely to receive compassion from others, whilst higher social safeness also predicted compassion from others in Sri Lankan participants.

**Table 6.** Regression results for predictors of compassion from others in Sri Lankan participants

Block 3	*B*	SEB	*β*	*t*	Sig.	*r* zero order	sr^2^	95% CI
Buddhist religion	2.53	3.30	0.07	0.77	0.444	0.10	0.003	−3.99 to 9.06
Atheist religion	3.18	3.59	0.08	0.89	0.377	−0.05	0.004	−3.92 to 10.27
**Gender**	**7.61**	**2.96**	**0.22**	**2.57**	**0.011**	**0.16**	**0.038**	**1.76 to 13.45**
Age	0.04	0.30	0.01	0.12	0.904	−0.06	0.000	−0.55 to 0.62
Anxiety	−0.52	0.37	−0.16	−1.42	0.157	−0.13	0.012	−1.24 to 0.20
Depression	0.47	0.34	0.19	1.36	0.177	−0.12	0.011	−0.21 to 1.14
FCTO	0.05	0.24	0.02	0.21	0.832	0.06	0.000	−0.42 to 0.52
FCFO	−0.06	0.25	−0.04	−0.25	0.804	−0.11	0.000	−0.56 to 0.43
FSC	0.16	0.19	0.13	0.84	0.403	−0.08	0.004	−0.21 to 0.53
Inadequate self	0.06	0.29	0.03	0.21	0.831	−0.15	0.000	−0.51 to 0.63
Reassured self	0.01	0.33	0.01	0.04	0.971	0.24	0.000	−0.64 to 0.67
Hated self	−0.01	0.57	−0.00	−0.02	0.988	−0.18	0.000	−1.13 to 1.11
Others as shamer	−0.04	0.14	−0.04	−0.26	0.794	−0.18	0.000	−0.31 to 0.24
Social safeness	0.82	0.20	0.48	3.98	0.000	0.39	0.092	0.41 to 1.23

*Note.* sr^2^: small effect size = 0.02, medium effect size = 0.15, large effect size = 0.35.

Variable that has a significant *p* value of (<.05) has been presented in bold.

A significant regression equation [*F*(14, 284) = 15.54, *p* < 0.001] with an *R*^2^ of 0.43 was found for compassion from others (Table 7) in UK participants. Being younger, lack of fear of receiving others' compassion, low external shame, lower depression, higher social safeness and higher anxiety all predicted compassion from others, in the UK participants (Tables 6 and 7).

**Table 7.** Regression results for predictors of compassion from others in UK participants

Block 3	*B*	SEB	*β*	*t*	Sig.	*r* zero order	sr^2^	95% CI
Buddhist religion	−4.56	6.08	−0.04	−0.75	0.454	−0.06	0.001	−16.53 to 7.40
Atheist religion	−0.05	0.71	−0.00	−0.06	0.950	−0.02	0.000	−1.45 to 1.36
Gender	1.55	2.42	0.03	0.64	0.521	0.08	0.001	−3.21 to 6.31
**Age**	−**0.41**	**0.12**	−**0.17**	−**3.49**	**0.001**	−**0.30**	**0.024**	−**0.64 to −0.18**
**Anxiety**	**0.69**	**0.22**	**0.24**	**3.19**	**0.002**	−**0.25**	**0.020**	**0.27 to 1.12**
**Depression**	−**0.55**	**0.20**	−**0.23**	−**2.70**	**0.007**	−**0.43**	**0.015**	−**0.94 to −0.15**
FCTO	−0.04	0.12	−0.02	−0.32	0.750	−0.24	0.000	−0.28 to 0.20
**FCFO**	−**0.37**	**0.13**	−**0.25**	−**2.84**	**0.005**	−**0.51**	**0.016**	−**0.63 to −0.11**
FSC	0.14	0.09	0.12	1.48	0.139	−0.37	0.004	−0.05 to 0.32
Inadequate self	−0.03	0.17	−0.02	−0.18	0.854	−0.36	0.000	−0.36 to 0.30
Reassured self	0.09	0.18	0.04	0.49	0.624	0.38	0.000	−0.26 to 0.44
Hated self	0.33	0.24	0.11	1.35	0.179	−0.35	0.004	−0.15 to 0.80
**Others as shamer**	−**0.16**	**0.08**	−**0.16**	−**2.02**	**0.044**	−**0.46**	**0.008**	−**0.32 to −0.00**
**Social safeness**	**0.59**	**0.12**	**0.36**	**4.95**	**0.000**	**0.57**	**0.049**	**0.36 to 0.83**

*Note.* sr^2^: small effect size = 0.02, medium effect size = 0.15, large effect size = 0.35.

Variable that has a significant *p* value of (<.05) has been presented in bold.

## Discussion

This study investigated the differences and similarities between the three flows of compassion (self-compassion, compassion to others and compassion from others), and inhibitors (fear of self-compassion, fear of compassion to others, fear of compassion from others, self-criticism and external shame), facilitators of compassion (self-reassurance and social safeness) and psychopathology (depression and anxiety) between a cross-cultural sample of Sri Lankan and UK participants. In comparison with the UK participants, Sri Lankan participants indicated higher levels of self-compassion, self-reassurance, external shame and fears of compassion (when controlling for age and gender). In contrast, the UK participants reported higher social safeness.

This study also explored individual predictors of the three flows of compassion for each country, as this may be helpful in adapting interventions with cultural sensitivity. When controlling for religion, age, gender, anxiety and depression, some similar and some different predictors were identified for each flow of compassion, as discussed below.

### Self-compassion

The significantly higher levels of self-compassion in Sri Lankans may be explained by the fact that 74% of them were Buddhists, compared to the 1% in the UK group, in which 62% of the UK participants self-identified as atheists. When testing the second hypothesis, the multiple regressions also indicated that following Buddhism strongly predicted self-compassion in Sri Lankans. Buddhist compassion teaches that one should fully cultivate self-compassion, prior to practising it on others (Salzberg, 2012; Bhikkhu, 2018). Thus, this study suggested that the strong Buddhist influence on self-compassion might at least partially explain the cross-cultural difference in the two groups. In addition to following Buddhism, older age and lack of fear of self-compassion predicted self-compassion in Sri Lankan participants. Hwang *et al*. (2016) found that middle-aged adults, as compared to young adults in Japan, practiced self-compassion as a more vital construct towards leading a prosperous and psychologically healthy life.

Similar to the current study, Steindl *et al*. (2020) also found higher self-compassion in the Singaporean sample compared to the Australian sample. Although one would presume people from individualistic backgrounds (e.g. Australia) to be more self-compassionate (Steindl *et al*., 2020), Montero-Marin *et al*. (2018) emphasised that the individualistic social dynamic could suppress self-compassion due to the high levels of competition-based motives, social comparisons and possibly higher self-criticism. Gilbert *et al*. (2017) also found that self-compassion and self-reassurance were significantly higher in a collectivistic Portuguese student sample in comparison with the UK and USA student samples. In consideration of individualistic communities, a study conducted among the UK students found that some participants perceived self-compassion as a self-indulgent construct (Gilbert *et al*., 2011). Thus, prospective research should explore whether the lower self-compassion in the UK group was due to a belief that self-compassion should not be in one's best interest (Robinson *et al*., 2016).

Interestingly, self-reassurance predicted self-compassion among participants in both countries. Whilst compassion is a sensitivity to suffering with the motivation to relieve that suffering, self-reassurance possesses the ability to soothe or reassure oneself during times of distress (Gilbert *et al*., 2004). Thus, self-compassion and self-reassurance have indicated strong correlations (Hermanto and Zuroff, 2016), which is unsurprising as self-reassurance buffers depression and self-criticism, both of which have shown negative correlations with self-compassion (Petrocchi *et al*., 2019). In support, Gilbert *et al*. (2017) found that self-compassion and self-reassurance were significantly higher in a Portuguese sample, which also indicated the lowest depression and anxiety scores in comparison with the UK and USA samples.

The results of this study indicated that in the UK group, higher perceived shame predicted higher self-compassion. This positive relationship is theoretically contradicting as the literature suggests that one's experiences of themselves as living negatively in the minds of others (external shame) is strongly correlated with low self-compassion and increased psychopathology (Ferreira *et al*., 2013). Therefore, in the context of societal shame, people may internalise that shame and become more self-critical as opposed to being self-compassionate (Matos *et al*., 2017). It was surprising that the UK participants' self-compassion was predicted by higher perceived external shame. Thus, further exploratory mediational analyses were conducted, which suggested that the higher self-reassurance in fact, explains how the negative direction between shame and self-compassion can turn into a positive relationship. This is further evidence highlighting the potential vital role of self-reassurance, even in the presence of external shame, as a mechanism to increase self-compassion. The SMT (Gilbert, 2014*b*) also details that self-reassurance activates a self-to-self caregiving mentality during times of distress, which in turn encourages people to direct compassion inwardly towards themselves.

### Compassion to others

Asian cultures are rich in interpersonal connectedness, social conformity and caring for others, compared to Western societies, such as the UK, that encourage individuality and autonomy (Markus and Kitayama, 1991; Gardner *et al*., 1999). Thus, one would expect Sri Lankans to be more compassionate towards others, given that they were also more self-compassionate. However, there was no significant difference in the levels of compassion to others between the two groups. Steindl *et al*. (2020) also found that Australians were more compassionate towards others compared to the Singaporeans, who come from a collectivistic society. Although they expected that the collectivistic social dynamic would encourage compassion towards others, the results led them to believe that the compassion offered in such cultures may be ‘submissive’ than ‘genuine’. In other words, when the compassion is referred to as submissive, it implies that the motive of the compassion given is based on obligation or submission, and possibly due to a fear of not being liked or valued if the compassion is not offered (Catarino *et al*., 2014). Sri Lankans in this study also indicated higher fears of compassion to others which may explain the non-significance in the results.

Contradictorily, however, Gilbert *et al*. (2017) found that Portuguese participants from a collectivistic background indicated significantly higher levels of compassion across all three flows of compassion, which was also reflected in their significantly low levels of depression and anxiety, compared to participants from the UK and USA samples. Although the three flows of compassion were related in Gilbert *et al*.'s study, other studies indicated that self-compassion and compassion to others may not be correlated (Neff and Pommier, 2013; López *et al*., 2018) and that self-compassion is independent from developing compassion towards others (Abele and Wojciszke, 2007).

In the current study, Sri Lankans' likelihood of compassion towards others was predicted by greater fear of self-compassion and self-inadequacy. It is possible that people who feel inadequately about themselves have greater sympathy for the suffering of others and therefore, develop more compassion, in the same way having high anxiety is linked to developing sympathetic considerations towards others (Gambin and Sharp, 2016). This may also explain why Sri Lankans expressed compassion to others, even when they were fearful of showing themselves compassion. Cross cultural studies have identified compassion to others as a submissive function in Asian people (Catarino *et al*., 2014), and implied that people submissively show compassion to others, in order to avoid being rejected, although this may not increase their life satisfaction (Asano *et al*., 2020). Thus, a plausible explanation of fear of self-compassion predicting higher compassion towards others in the Sri Lankan group may be that, despite the fear of treating themselves with compassion, they may have felt compelled to offer it to others, to avoid social rejection. Previous studies identified that some Sri Lankans offer compassion to others, out of obligatory and submissive reasons (Kariyawasam *et al*., 2021).

In the UK participants, higher anxiety, being female and lack of fear of compassion to others predicted compassion towards others. In consideration of the gender difference, Western studies (Sprecher and Fehr, 2005) found that the nurturing and caring tendencies in females increased their compassion to others. Fear of compassion is known to inhibit compassion and stem from insecure attachments with others (Gilbert *et al*., 2011). Thus, it is unsurprising that the lack of fear predicted compassion to others in the UK participants, especially given that they reported higher social safeness.

### Compassion from others

As with offering compassion, Sri Lankan participants were expected to experience higher compassion from others (Markus and Kitayama, 1991; Pathirana, 2016). However, there was no significant difference in compassion from others between the two groups. Steindl *et al*. (2020) found higher compassion from others in Australian participants compared to that in Singaporean participants, concluding that the collectivistic nature and the perception of help seeking behaviour as being weak or shameful in the Asian communities may have resisted the Singaporean participants from seeking help or being open to receiving compassion from others. However, Gilbert *et al*. (2017) found highest compassion from others in a Portuguese sample when compared to the UK and USA populations. Although Portugal is considered to have a collectivistic culture (Hofstede, 2011), the higher density of the ‘shame’ component in Asian countries should be explored further, to determine to understand this cultural distinction.

Social safeness predicted compassion from others in both groups. Previous studies also propose that social safeness mediates the capacity to receiving compassion (Kelly and Dupasquier, 2016) and that the lack of social safeness increases trust issues and the perception that others are judgemental and rejecting (Gilbert, 2014*a*). Thus, results suggest that participants perceived others as compassionate givers when they felt safe within their social relationships, as social safeness increases affiliative interactions with others (Kelly and Dupasquier, 2016), which in turn activates the soothing system (Gilbert, 2014*a*).

Furthermore, being younger in age predicted compassion from others in the UK participants, together with high levels of anxiety, low levels of depression, lack of external shame and lack of fear of receiving compassion from others. It feels fair to address whether younger participants may have experienced stronger parental attachments and connections with the society, which may explain the higher perception of compassion from others. In fact, warm parental relationships enhance the soothing system and increase social safeness (Cacioppo *et al*., 2000). Future studies on student populations should, therefore, investigate participants' relationship with their parents, for a better understanding of this phenomenon. The current study also noted that participants who were less depressed were more likely to perceive higher compassion from others. Self-critical and depressed people tend to show a lack of ability to receive affection and compassion from others (Bowlby, 1982) and resist compassion, even when it is offered (Gilbert and Procter, 2006). This also implies that people who are depressed and self-critical may perceive others as not compassionate as a way of resisting compassion from others. Anxiety is positively associated with affective empathy and sympathy (Gambin and Sharp, 2016), indicating that people with anxiety may also have increased considerations towards the suffering of others.

Another cultural difference was that being female in the Sri Lankan group, compared to males, was a predictor of compassion from others, which is supported by females' natural propensity towards engaging compassionately with others (Stellar *et al*., 2012; Neff and Pommier, 2013). It is noteworthy that there was no gender difference in the UK group, which raises the question whether the autonomous social background may have prevented UK females from seeking compassion, leading them to believe that others are not compassionate towards them. In fact, distance is identified as a positive cultural value in the UK, which links with respect for individual autonomy (Paxman, 2007). The impact of autonomy on seeking compassion, therefore, needs further exploration.

### Strengths and limitations

This was the first cross-cultural study to investigate the three flows of compassion in a Sri Lankan sample in comparison with a UK sample. This was also the first study exploring Gilbert's (2014*a*) SMT in a Sri Lankan population using a questionnaire-based approach. The use of a series of validated measures and the detailed explanation of the nuances of the three flows of compassion further strengthened the overall study quality. This study also contributed to the understanding of the seemingly strong influence of Buddhism on self-compassion, signifying the importance of further explorations to integrate these vital elements when conducting compassion-based interventions. Additionally, future research may incorporate measures to investigate the associations between religion, culture, well-being and the different components of compassion to further understand these factors as potential facilitators/inhibitors of compassion.

In consideration of the weaknesses, as this study was cross-sectional, drawing conclusions on the causality of compassion, inhibitors and facilitators was problematic (Matos *et al*., 2017). Importantly, as data collection continued during the COVID-19 pandemic (Jia *et al*., 2020), it is possible that compassion levels varied from the usual levels in all participants, especially in the UK group due to the high-pandemic impact at the time of data collection. This may also explain the non-significance in depression and anxiety between the two countries. Although studies have found people in collectivistic countries to be more distressed than people in the West (Birkett, 2013), the alarming situation of the pandemic in the UK might suggest that the UK participants' depression and anxiety levels may have been higher than usual. In fact, a UK study found that during the pandemic, depression and anxiety exceeded the population norms especially among young people (Jia *et al*., 2020), who comprised most of the UK sample of this study.

Although religion was accounted as a possible variable of cross-cultural differences observed in the results, this study did not assess differences in cultural or group norms (e.g. collectivistic/individualistic), and other cultural practices which may have had an impact on the overall study results. Furthermore, the study was mostly advertised among university students, limiting the sample to young people in both countries which limits the generalisability of this research. This study was also conducted in English language, allowing only English-speaking Sri Lankans to participate.

### Clinical implications and recommendations for future research

This study aimed to facilitate an enhanced cross-cultural understanding of compassion across its three flows (Gilbert *et al*., 2017), which is predominantly a Western approach. Considering the impact of compassion-based approaches (e.g. CFT) on reduced levels of anxiety and depression in Western communities (Gilbert *et al*., 2011), the application of these approaches cross-culturally (e.g. Sri Lanka) may result in increased well-being in people around the world. The findings tentatively suggest that compassion-based trainings may be helpful, but also identified that cultural differences should be considered when tailoring individual treatments. It appears that religion, Buddhism in particular in this study, and other demographic factors should be accounted for. This is vital in therapeutic contexts as these may be useful protective factors in enhancing a person's well-being. When implementing interventions such as CFT, clinicians should also address inhibitors such as fears of offering and receiving compassion, and negative shame-based emotional experiences (such as self-criticism and external shame) stimulating such fears, in order to assist self-generating compassion and reception towards compassion in clients (Matos *et al*., 2017).

## Conclusion

This study identified that cultural differences and similarities were present between UK and Sri Lankan participants in their levels of self-compassion, compassion to and from others. Sri Lankans were significantly more self-reassured and self-compassionate, although they also reported higher external shame and fears of compassion. UK participants found more safeness in others, despite their individualistic social dynamic (Gardner *et al*., 1999). Regardless of the cultural differences, those who felt highly self-reassured were more self-compassionate. Buddhism predicted greater self-compassion in Sri Lankans although external shame and insecure attachments inhibited their compassionate experiences with others. Overall, this study signified the importance of paying close attention to cultural and religious influences when exploring compassion across cultures. Irrespective of the individualistic-independent and collectivistic-interdependent cultural context, this study highlighted the potential role that significant others and society play in one's level of compassion and well-being. As compassion cultivation across all three flows has resulted in increased well-being and reduced psychological distress (Gilbert, 2005*a*), it is vital that differences between countries are considered when introducing Western psychotherapeutic approaches into non-Western settings such as Sri Lanka.
